# Protective Effect of *Salicornia europaea* Extracts on High Salt Intake-Induced Vascular Dysfunction and Hypertension

**DOI:** 10.3390/ijms17071176

**Published:** 2016-07-21

**Authors:** Nisha Panth, Sin-Hee Park, Hyun Jung Kim, Deuk-Hoi Kim, Min-Ho Oak

**Affiliations:** 1 College of Pharmacy and Natural Medicine Research Institute, Mokpo National University, Muan-gun, Jeonnam 58554, Korea; panth.nisha1@gmail.com (N.P.); sin-hee.park@unistra.fr (S.-H.P.); hyunkim@mokpo.ac.kr (H.J.K.); 2 UMR CNRS 7213, Laboratoire de Biophotonique et Pharmacologie, Faculté de Pharmacie, Université de Strasbourg, Illkirch 67401, France; 3 Research Center, Phyto Corporation, Seoul 08826, Korea; dhkim@phytoco.com

**Keywords:** *Salicornia europaea*, vascular dysfunction, blood pressure, hypertension

## Abstract

High salt intake causes and aggravates arterial hypertension and vascular dysfunction. We investigated the effect of *Salicornia europaea* extracts (SE) on vascular function and blood pressure. SE constituents were analyzed using high performance liquid chromatography, and SE’s effect on vascular function was evaluated in isolated porcine coronary arteries. SE’s vascular protective effect was also evaluated in vivo using normotensive and spontaneous hypertensive rats (SHRs). SE mainly contained sodium chloride (55.6%), 5-(hydroxymethyl)furfural, *p*-coumaric acid, and *trans*-ferulic acid. High sodium (160 mmol/L) induced vascular dysfunction; however, SE containing the same quantity of sodium did not cause vascular dysfunction. Among the compounds in SE, *trans*-ferulic acid accounts for the vascular protective effect. Normotensive rats fed a high-salt diet showed significantly increased systolic blood pressure (SBP), diastolic blood pressure (DBP), and mean arterial pressure (MAP), which decreased significantly in the SE-treated groups. In SHRs, high edible salt intake significantly increased SBP, DBP, and MAP, but SE intake was associated with a significantly lower MAP. Thus, SE did not induce vascular dysfunction, and *trans*-ferulic acid might be at least partly responsible for the vasoprotective effect of SE. Taken together, SE could be used as an alternative to purified salt to prevent and ameliorate hypertension.

## 1. Introduction

Cardiovascular disease (CVD) is one of the leading causes of disability and death worldwide. In 2013, the World Health Organization (WHO) reported that high blood pressure (BP) is common and the most important risk factor of CVD, and nearly 40% of the world’s adult population has hypertension. The prevalence of hypertension is increasing rapidly, and several epidemiological studies have reported that it would escalate by 24% in developed countries and 80% in developing countries by 2025 [1,2]. The majority, 90%–95% of individuals with hypertension, are classified as having “essential” hypertension, wherein the medical establishment claims that the cause is idiopathic or unknown [3,4]. Hypertension is influenced by both genetic and environmental factors. Among the environmental factors, salt is the major factor that increases blood pressure and salt intake-induced elevation of blood pressure is a major public health challenge [5]. Although dietary sodium is a well-known essential electrolyte that plays an important role in the regulation of body fluids and the cardiovascular system, the physiological need of sodium is only around 1.2 g/day. However, the global salt intake is much higher at approximately 9 g/day [6]. Despite salt’s important role in the body, excessive consumption of salt is deleterious for health, mainly because it triggers, at least in part, the development of essential hypertension and related cardiovascular complications [7,8].

These days, researchers are extensively studying several naturally occurring traditional medicines for preventing hypertension [9,10,11]. *Salicornia europaea* is a well-known bioactive halophyte that grows in high-salt coastal marshes, including those along the western coast of South Korea. *S. europaea* has been used as a folk medicine to treat various kinds of diseases such as cephalalgia and scurvy [12], and is consumed in a variety of ways in the coastal areas of Korea. The use of the plant extract has been recently extended to the development of functional foods to maintain the health of the cardiovascular system, especially as a substitute for purified salt. *S. europaea* is rich in salt and minerals such as magnesium, calcium, potassium, and iron; therefore, it is suitable for developing a purified salt substitute [13]. However, the effect of *S. europaea* extracts (SE) on vascular dysfunction and blood pressure in comparison with those of highly purified salt remains unclear. Therefore, in this study, we investigated whether intake of SE can influence the function of coronary arteries and blood pressure in an animal model, especially in comparison with highly purified salt.

## 2. Results

### 2.1. Composition of Salicornia europaea Extracts (SE)

**Figure 1:**
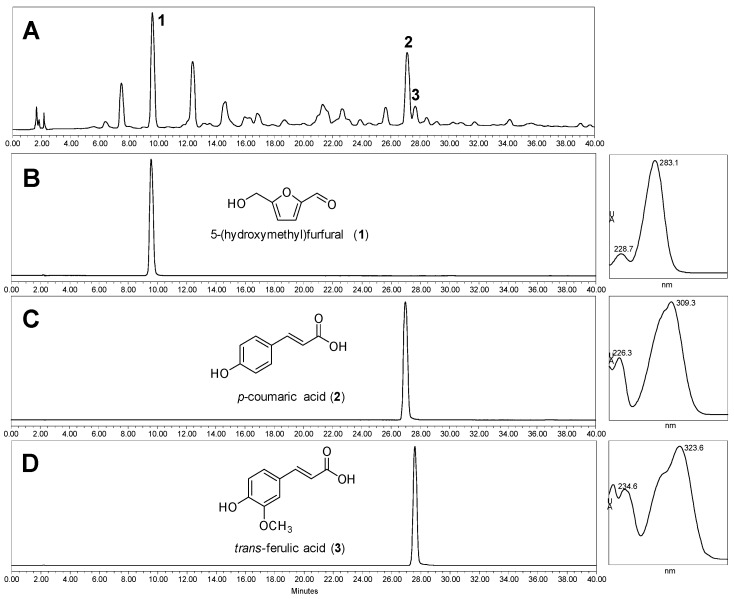
HPLC profiles for *Salicornia europaea* extracts (SE) and three reference compounds: 5-(hydroxymethyl)furfural (1); *p*-coumaric acid (2); and *trans*-ferulic acid (3). Chromatograms were extracted at 305 nm. (**A**) chromatograms of SE; and (**B**–**D**) HPLC profile and UV spectra of 5-(hydroxymethyl)furfural (1); *p*-coumaric acid (2); and *trans*-ferulic acid (3).

The composition analysis indicated that the main nutrients and salt contents of SE were as follows: NaCl, 55.6%; carbohydrate, 17.9%; protein, 9.8%; fat, 0.4%; potassium, 3.1%; calcium, 0.7%; and magnesium, 1%. SE analysis by high-pressure liquid chromatography (HPLC) indicated the presence of three compounds: 5-(hydroxymethyl)furfural (1); *p*-coumaric acid (2); and *trans*-ferulic acid (3), with retention times (*t*_R_) of 9.61, 27.12, and 27.68 min, respectively (Figure 1). The *t*_R_ values and ultra violet (UV) spectra obtained were identical to those obtained for reference compounds under the same HPLC experimental conditions. Contents of compounds **1**–**3** in SE were determined by HPLC using a reverse phase system eluted with a gradient solvent profile consisting of 0.1% formic acid-containing water and acetonitrile i.e., acetonitrile 2% to 12% linearly at 0–6 min; 12% to 35% at 6–7 min; and 35% to 50% at 7–15 min. The regression equation and correlation factor of each calibration curve were as follows: 5-(hydroxymethyl)furfural, *y* = 15.760*x* + 6.6485 (*r*^2^ = 0.9990); *p*-coumaric acid, *y* = 51.162*x* − 1.0993 (*r*^2^ = 0.9998); *trans*-ferulic acid *y* = 26.682*x* − 1.7212 (*r*^2^ = 0.9998). As a result, contents of 5-(hydroxymethyl)furfural, *p*-coumaric acid, and *trans*-ferulic acid in SE were finally determined as 18.20 ± 1.80, 3.19 ± 0.47, and 2.60 ± 0.33 µg/g, respectively.

### 2.2. Effect of SE on High Salt-Induced Vascular Dysfunction in Porcine Coronary Arteries

**Figure 2:**
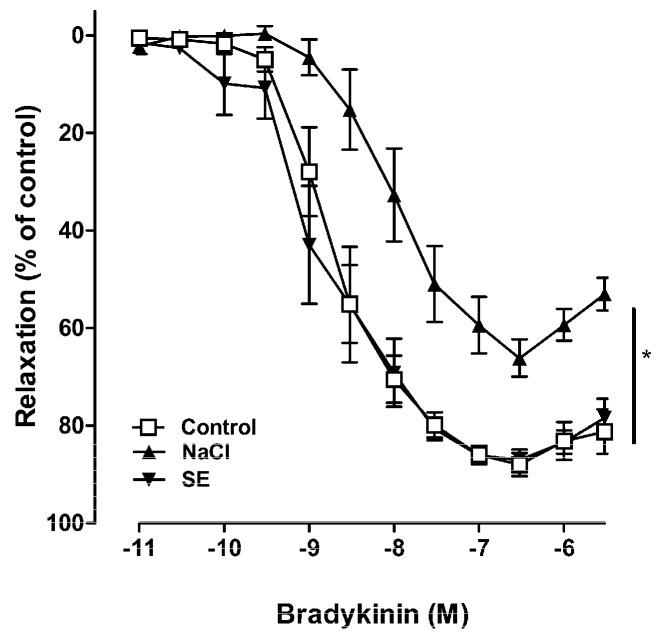
Relaxant responses to bradykinin in porcine coronary arteries following incubation for 18 h with a medium containing a high sodium concentration (160 mmol/L) or *Salicornia europaea* extracts (SE) at the concentration equivalent to 160 mmol/L sodium. Relaxant responses are expressed as the percentage of relaxation of the initial tone induced by U46619. Values are expressed as mean ± SEM from *n* = 6 to 10 experiments. Concentration-response curves were compared using two-way ANOVA. “*” stands for *p* < 0.05 vs. control.

**Figure 3:**
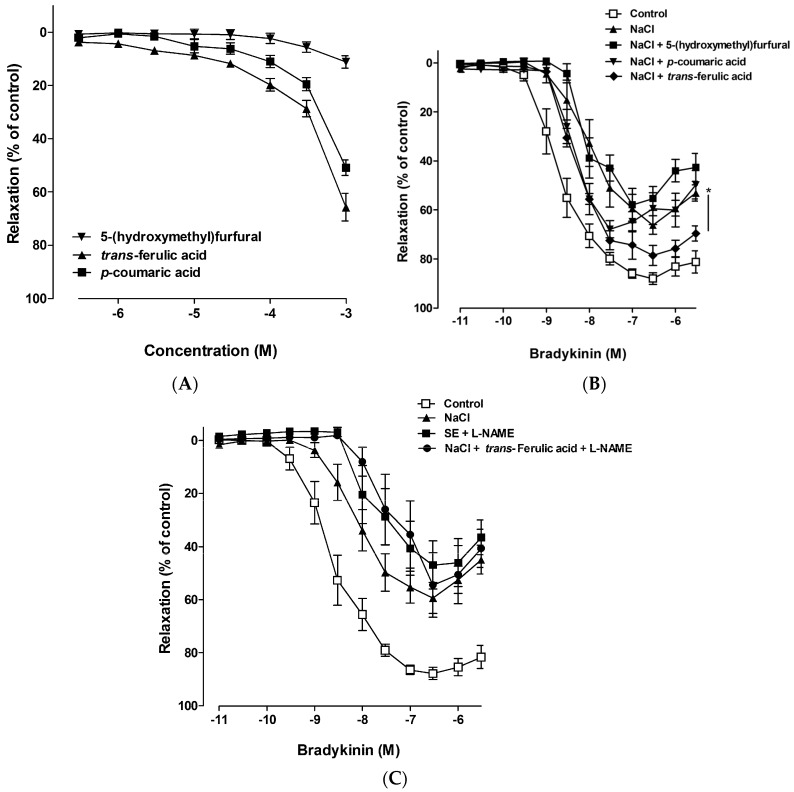
Dose-dependent vasorelaxant effects of 5-(hydroxymethyl)furfural, *p*-coumaric acid, and *trans*-ferulic acid in porcine coronary arteries (**A**) and vasorelaxant responses to bradykinin in porcine coronary arteries following incubation for 18 h with a medium containing a high sodium concentration (160 mmol/L) in presence or absence of 5-(hydroxymethyl)furfural (0.1 mmol/L), *p*-coumaric acid (0.1 mmol/L), and *trans*-ferulic acid (0.1 mmol/L) (**B**) and pre-treated with L-NAME (an inhibitor of eNOS, 0.1 mmol/L) (**C**). Relaxant responses are expressed as the percentage of relaxation of the initial tone induced by U46619. Values are expressed as mean ± SEM from *n* = 8 to 10 experiments. Concentration-response curves were compared using two-way ANOVA. “*” stands for *p* < 0.05 vs. NaCl.

In porcine coronary arteries, bradykinin produced a relaxing effect, which was mediated by endothelial nitric oxide synthase (eNOS)-derived NO [14]. To test whether high salt induced vascular dysfunction or not, porcine coronary artery rings were incubated in a culture medium with high salt. The concentration of sodium in the Dulbecco Modified Eagle medium (DMEM) was 133 mM; therefore, a medium with high NaCl was prepared by adding NaCl and SE at the final concentration of 160 mmol/L sodium. The degree of relaxation induced by bradykinin was dose-dependent. The maximal relaxation was 87.96% ± 2.48% in the control coronary artery, whereas it was significantly reduced by pretreatment with high salt (160 mmol/L sodium, maximal response was 65.21% ± 3.76%) for 18 h (Figure 2). Despite treatment with the same amount of SE as the salt concentration, equivalent to 160 mmol/L of sodium, SE did not mediate vascular dysfunction. These results suggested that high salt induced vascular dysfunction, which was prevented by components in SE. Therefore, experiments were performed to isolate single compounds from SE, and to identify the active compound. Analysis of SE led to the identification of three major known compounds and some unknown compounds (Figure 1). The vascular reactivity study indicated that *p*-coumaric acid and *trans*-ferulic acid alone induced vasorelaxation in a dose-dependent manner in the coronary artery (Figure 3A); however, this vasodilation effect was remarkable only at high concentrations. However, vascular dysfunction induced by high salt was strongly ameliorated by treatment with *trans*-ferulic acid, not by 5-(hydroxymethyl)furfural or *p*-coumaric acid (Figure 3B). Since previous investigations have shown that ferulic acid enhanced vasodilation through an NO-mediated mechanism [15], the role of this pathway in the vasoprotective effects of SE and ferulic acid was determined. Pre-treatment with the eNOS inhibitor, L-NAME, blocked the vasoprotective effect of SE and ferulic acid (Figure 3C). Taken together, these results suggested that SE ameliorated high salt-induced vascular dysfunction via the eNOS pathway and its protective effect is attributable to its component, *trans*-ferulic acid.

### 2.3. Influence of High Salt and SE on Body Weight and Food Consumption

**Figure 4:**
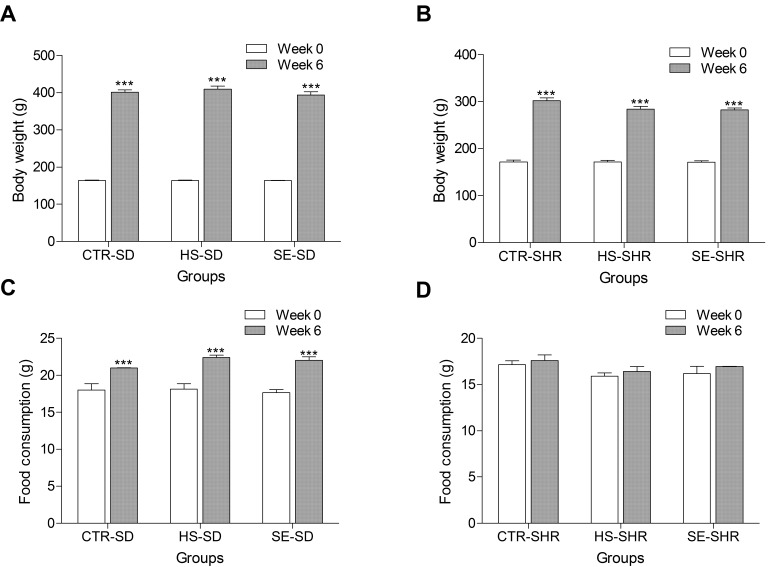
Body weight (**A**,**B**) and food consumption (**C**,**D**) were examined in spontaneously hypertensive rats (**B**,**D**) and Sprague–Dawley (**A**,**C**) rats fed high edible salt and *Salicornia europaea* extracts (SE) for six weeks. CTR–SD, control SD rat; HS–SD, high edible salt-fed SD rat (800 mg/kg/day); SE–SD, SE-fed SD rat (1400 mg/kg/day); CTR–SHR, control SHR; HS–SHR, high edible salt-fed SHR (800 mg/kg/day); SE–SHR, SE-fed SHR (1400 mg/kg/day). Values are expressed as mean ± SEM. “***” stands for *p* < 0.001 vs. week 0.

To confirm the vascular protective effect of SE, an in vivo study was performed using normotensive and hypertensive animals respectively. Young spontaneous hypertensive rats (SHR) and age-matched Sprague–Dawley rats (SD) were randomly divided into six groups: G1, vehicle control group (CTR–SD); G2, vehicle control group (CTR–SHR); G3, high salt (NaCl 800 mg/kg/day group, HS–SD); G4, high salt (NaCl 800 mg/kg/day group, HS–SHR); G5, SE 1400 mg/kg/day group (SE–SD); and G6, SE 1400 mg/kg/day group (SE–SHR). All groups showed an increase in body weight during the experimental period (Figure 4A,B). The cumulative body weight gain was less in the SHR groups than in the SD groups; this finding was consistent with those reported previously [16,17,18,19]. This change might be, at least in part, due to lower food intake in the SHR groups because the total daily food intake was significantly lower in the SHR groups (CTR–SHR, 17.58 ± 0.62 g; HS–SHR, 16.41 ± 0.53 g; SE–SHR, 16.95 ± 0.03 g) than in the SD groups (CTR–SD, 21.01 ± 0.01 g; HS–SD 22.41 ± 0.3 g; SE–SD 22.00 ± 0.5 g) (Figure 4C,D). In contrast, no differences in body weight gain and food intake were observed between the high salt-fed rat groups and SE-fed rat groups for any strain.

### 2.4. Effect of SE on Blood Pressure in Normotensive Rat

**Figure 5:**
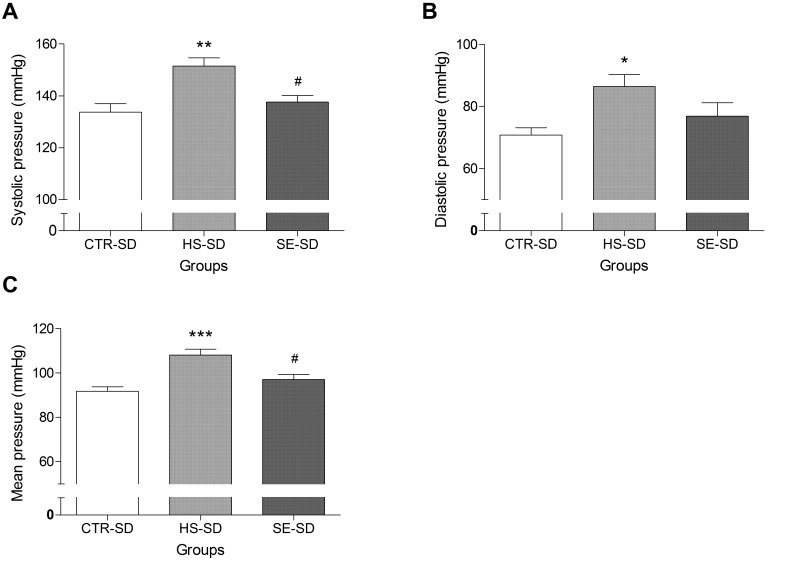
Effect of *Salicornia europaea* extracts (SE) on the systolic blood pressure (**A**); diastolic blood pressure (**B**); and mean arterial pressure (**C**) in a normotensive SD rat group (CTR–SD) compared with the values in a high edible salt-fed group (HS–SD, 800 mg/kg/day) and a group fed SE (SE–SD, 1400 mg/kg/day). Values are expressed as mean ± SEM. “*”, “**” and “***” stand for *p* < 0.05, *p* < 0.01, and *p* < 0.001 vs. CTR–SD, respectively. “#” stands for *p* < 0.05 vs. HS–SD.

During adaptive feeding, blood pressure in the SD rat groups did not differ significantly. A high NaCl diet (HS-SD group) for six weeks caused a significant increase in systolic blood pressure (SBP), diastolic blood pressure (DBP), and mean arterial pressure (MAP), in comparison with the values in the untreated control group (CTR–SD). However, SBP (HS–SD: 151.49 ± 3.11 vs. SE–SD: 137.56 ± 2.50 mmHg) and MAP (HS–SD: 108.11 ± 2.59 vs. SE–SD: 97.04 ± 2.31 mmHg) of the SE-treated group were significantly lower than those of the high NaCl-treated group were (Figure 5A,C). The DBP after SE treatment was slightly lower than that after high NaCl intake, but the difference was not significant (Figure 5B). This result suggested that highly purified salt intake had a significant effect on blood pressure while SE, which has the same high levels of NaCl, had little impact on blood pressure in a normotensive rat.

### 2.5. Effect of SE on Blood Pressure in Hypertensive Rats

**Figure 6:**
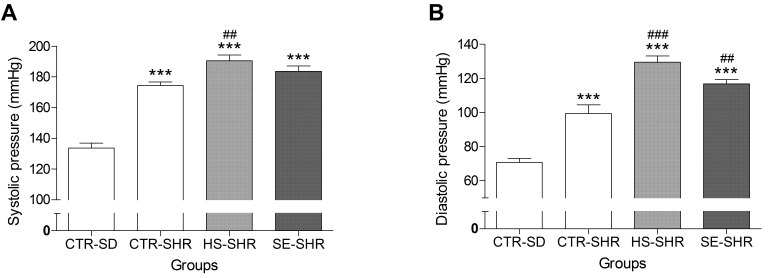

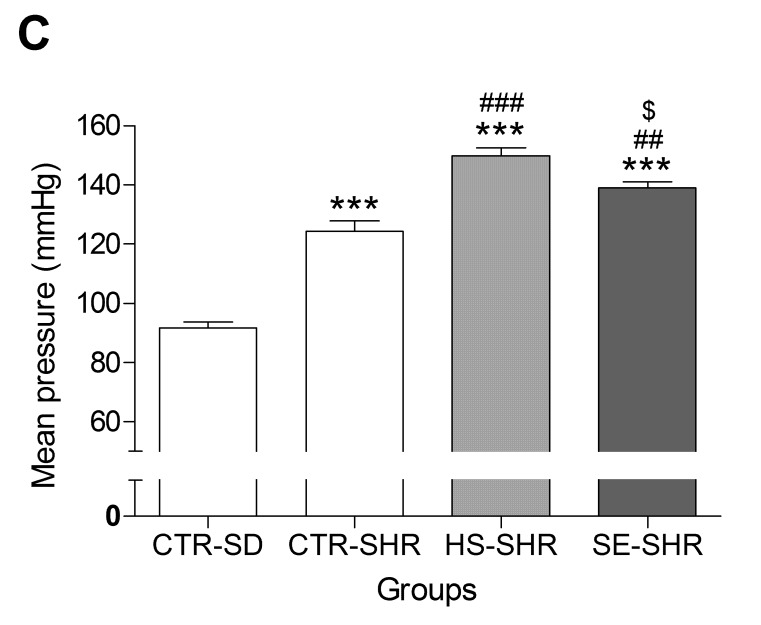
Effect of *Salicornia europaea* extracts (SE) on the systolic blood pressure (**A**); diastolic blood pressure (**B**); and mean arterial pressure (**C**) in the spontaneous hypertensive rat group (CTR–SHR) compared to the values in the high edible salt fed group (HS–SHR, 800 mg/kg/day) and the SE group (SE–SHR, 1400 mg/kg/day). Values are expressed as mean ± SEM. “***” stands for *p* < 0.001 vs. CTR-SD. “##” and “###” stands for *p* < 0.01 and *p* < 0.001 vs. CTR–SHR respectively. “$” stands for *p* < 0.05 vs. HS–SHR.

At six weeks of age, the SBP, DBP, and MAP of all SHR groups (CTR–SHR, HS–SHR, and SE–SHR) were significantly higher than those of the CTR–SD group were (Figure 6A–C). The SBP, DBP, and MAP of the HS–SHR group were significantly higher than those of the CTR–SHR group were, indicating that high salt intake exacerbates hypertensive status. The SBP and DBP of the SE treatment group were slightly lower than those of the high salt treatment group were, but the difference was not significant. However, the MAP in the SE-treated group was significantly (10 mmHg) lower than that in the high salt group (HS–SHR: 149.91 ± 2.73 vs. SE–SHR: 139.02 ± 2.36 mmHg). Taken altogether, these results suggest that highly purified salt intake increased blood pressure while SE, which has the same high levels of Na, had little impact on blood pressure not only in normotensive rats, but also in hypertensive rats.

### 2.6. Kidney Index and Cardiac Index

The kidney index (KI) of the HS group in both normotensive and hypertensive rats was slightly higher than that of the control group, but the difference was not significant (Table 1). Although the cardiac index (CI) of SHR groups was significantly higher than that of the CTR–SD group, there were no significant differences among the CTR, HS, and SE groups of SD and SHR.

## 3. Discussion

Daily moderate intake of salt (sodium chloride, NaCl) is essential to maintain proper homeostasis of extracellular fluid volume, blood pressure, and vascular function; however, excessive salt intake is known to lead to manifestation and aggravation of arterial hypertension and vascular dysfunction [16]. It was previously reported that high salt intake induces endothelial stiffening, resulting in vascular dysfunction via reduction of NO release [17,18]. Endothelial dysfunction is a pathological condition that is mostly characterized by an imbalance between vasodilator and vasoconstrictor substances, especially NO, and this imbalance leads to impairment of endothelium-dependent relaxation, a functional characteristic of vascular dysfunction [19]. Consistent with the findings of previous reports, our study showed that high salt intake induced vascular dysfunction; however, the SE did not induce vascular dysfunction, even when the salt concentration was the same as that in the high salt intake condition. This result suggested that some component in SE confers protective effects against high salt-induced vascular dysfunction. The composition analysis revealed that SE mainly contained 5-(hydroxymethyl)furfural, *p*-coumaric acid, *trans*-ferulic acid, and other unknown compounds. Among these, *trans*-ferulic acid is responsible for the vascular protective effect of SE. Although *p*-coumaric acid and *trans*-ferulic acid induced slight vasodilation by themselves (10.92% and 19.76% relaxation at 0.1 mmol/L, respectively), *trans*-ferulic acid significantly ameliorated the high salt-induced vascular dysfunction. Similarly, Suzuki et al. have reported that *trans*-ferulic acid restored endothelium-dependent vasodilation in the aortas of SHR, but showed no effect on aortas from normotensive Wistar Kyoto rats [15]. A previous report suggested that high salt consumption induces endothelial dysfunction with altered endothelial NO production [20], and *trans*-ferulic acid significantly potentiated the acetylcholine-induced vascular response of SHR aortas by enhancing the bioavailability of basal and stimulated NO and inhibiting NAD(P)H oxidase [15], a main source of ROS in the vasculature. Consistent with previous reports, pre-treatment with the eNOS inhibitor, L-NAME, blocked the vasoprotective effect of SE and *trans*-ferulic acid (Figure 3c). Although the exact underlying molecular mechanism of the protective effect of *trans*-ferulic acid and SE on high salt-induced vascular dysfunction remains to be investigated, especially in vivo effects on oxidative stress and inflammation, our results suggested that the increase of NO bioavailability by *trans*-ferulic acid might, at least in part, contribute to the vascular protective effect of SE. SE analysis by HPLC indicated that the contents of 5-(hydroxymethyl)furfural, *trans*-ferulic acid, and *p*-coumaric acid in SE were 18.20 ± 1.80, 2.60 ± 0.33, and 3.19 ± 0.47 μg/g, respectively. The concentration of ferulic acid used in vascular reactivity study was higher than its quantity in SE; this is to compensate for the loss of compounds from SE during extraction step for quantification due to the high quantity of salt in SE and also to determine whether *trans*-ferulic acid showed vasoprotective effects or not. Although *trans*-ferulic acid ameliorated high salt-induced vascular dysfunction, other unknown compounds in SE could also have vasoprotective effects or synergistic effects with ferulic acid; *trans*-ferulic acid might be not the only single active compound responsible for the vasoprotective effect of SE. In conclusion, full characterization of SE components needs to be performed to elucidate the underlying exact mechanism.

To confirm the vasoprotective effect of SE in comparison with high edible salt in vivo, we further investigated the effects of SE intake on blood pressure in normotensive and hypertensive animal models by comparing the findings with those associated with high intake of pure NaCl. The SE group of normotensive animals, which received the same amount of sodium as the HS group, exhibited a significant difference in SBP and MAP compared with the HS group. Moreover, there was no significant difference in SBP and DBP between the SE group and the control group. Thus, high-dose SE intake might prevent the increase in blood pressure to a certain extent while edible salt induces hypertension. In hypertensive animals, the HS–SHR group showed higher SBP and DBP than the CTR–SHR group, suggesting that highly purified sodium intake could exacerbate hypertensive status. However, the SE group showed no significant difference in SBP in comparison with the CTR–SHR group, but showed a significant difference in MAP when compared with the HS–SHR group. Taken together, these results suggest that although SE contained the same quantity of sodium as highly purified NaCl, intake of SE prevents the occurrence of hypertension and ameliorates the hypertensive status.

It is well known that high sodium intake triggers an increase in blood pressure, which is a major risk factor for cardiovascular disease. With the recent increase in the intake of dietary salt, the prevalence of hypertension is currently about 30% of the world population [21]. Researchers who gathered data on deaths in 66 different countries concluded that 2.2 million deaths per year worldwide were attributable to high sodium intake [22]. For these reasons, WHO recommendations as well as many national medical societies encourage reduction in daily sodium intake (NaCl less than 5 grams). However, several lines of experimental evidence suggest that many hypertensive patients still consume more salt than the level recommended by the guidelines [23,24]. Conventional salt that we consume comprises mainly NaCl (90%–99%), but rarely contains minerals that are beneficial to the health of humans. It had been previously confirmed that the natural plant salt obtained from saltwort (*S. europaea*) contains the desired amounts of NaCl and phytoorganic minerals with a strong salty taste that is comparable to common edible salt. Nutritional analysis showed that extracts from various *Salicornia* spp., including *S. europaea*, contain many substances, such as proteins, polysaccharides, organic nutrients, and minerals [12,13]. SE intake could, at least in part, suppress the high catabolism process. A *Salicornia*-derived salt substitute is nutritionally more beneficial than the normal salt and is suitable for development as a new plant salt. In the present study, we attempted to hypothesize whether substitution of normal salt with SE can prevent the risk of cardiovascular disease associated with high blood pressure. This study showed that the body weight increase in all groups was modest but consistent and statistically significant throughout the experimental period. Consistent with previous data, body weight gain was significantly less in the SHR group in comparison with the age-matched SD group [25]. SE–SHR showed no statistically significant impact on body weight. Previous studies confirmed that increased administration of NaCl produced a remarkable elevation in blood pressure in normal rats as well as in a hypertensive rat model. We were particularly interested in the SHR because they have numerous similarities to humans with essential hypertension. In this study, SBP and DBP were elevated in high NaCl-fed SD rats and SHR six weeks after the treatment began, which is consistent with previously reported findings [26,27]. However, high doses of SE indeed attenuated the blood pressure elevation in high salt-fed SHR and SD rats. Many researchers suggest that a hyperkinetic circulation, specifically an elevation of CI, may be an initiating factor in the development of essential hypertension [28]. However, in our experimental model, there was no significant difference in the CI between the SD and SHR groups. These results might be influenced by the relatively short duration of treatment; therefore, long-term treatment of more than three months may show the differences in the CI described in the previous report [28]. Further research is needed to understand the mechanisms involved in the interaction of SE and blood pressure, which will thereby allow successful therapeutic interventions for high salt-induced vascular dysfunction and hypertension.

## 4. Materials and Methods

### 4.1. Ethics Statement

This study conforms to the Guide of Care and the Use of Laboratory Animals published by the US National Institutes of Health (NIH publication No. 85-23, revised 1996), and the protocol was approved by the local ethics committee (KAMSI-IACUC 15-KE-101).

### 4.2. Plant Material and Analysis of SE Constituents by HPLC

Fresh *S. europaea* was collected from the western seashore of South Korea, cleaned with tap water, and then extracted with water by using a hydrothermal extractor (Cosmos 660, Kyungseo E&P, Seoul, Korea). The filtered supernatant of SE was purified using activated carbon (Norit, Cabot Corporation, Boston, MA, USA) for 30 min and spray-dried to yield a powder (Eyela SD-1000, Tokyo Rikakikai Co., Tokyo, Japan). According to the Korean Pharmacopeia, the indirect precipitation titration method was used to determine the sodium content. The nutritional components of SE were determined by biochemical analyses. All chemicals were purchased from Sigma Chemical Co., (St. Louis, MO, USA) unless specified.

An aliquot (3.05 g) of SE was suspended in 150 mL of distilled water and extracted using the same volume of ethyl acetate at room temperature. The acquired ethyl acetate layer was concentrated at reduced pressure to afford an extract (36 mg). To identify compounds in SE, the extract was used to prepare an HPLC sample (50 mg/mL) with methanol. Analytical HPLC experiments were performed to identify compounds in this extract by using a Waters HPLC instrument (Milford, MA, USA) comprising a 1525 binary pump, 2707 autosampler, and 2998 photodiode array detector. The sample (10 μL) was injected onto a Waters SunFire^TM^ C18 (Milford) (4.6 × 150 mm, 5 μm) column with a flow rate of 1.0 mL/min at ambient temperature. Three standard compounds (100 μg/mL solution with methanol) including 5-(hydroxymethyl)furfural (1) obtained from Acros Organics (Geel, Belgium), *p*-coumaric acid (2), and *trans*-ferulic acid (3) were eluted under identical conditions. HPLC profiles for SE samples were recorded using a photodiode array detector (210–400 nm), and retention time (*t*_R_) and UV spectrum for each peak were compared with those of the standards. To quantify compounds in SE, analytical HPLC experiments were performed by using an Agilent 1260 Infinity series (Waldbronn, Germany) comprising a quaternary pump, an autosampler, a column oven, and a photodiode array detector. The mobile phase was composed of 0.1% formic acid-containing water and acetonitrile, and the gradient elution was as follows: acetonitrile 2% to 12% (0–6 min), 12% to 35% (6–7 min), 35% to 50% (7–15 min). Each sample (2 μL) was injected onto a Thermo Acclaim^TM^ Rapid Separation Liquid chromatography (RSLC) (Sunnyvale, CA, USA) (2.1 × 100 mm, 2.2 μm) column at a flow rate of 0.3 mL/min at ambient temperature. HPLC profiles for reference compounds and SE samples were recorded using a photodiode array detector (210–400 nm), and retention time (*t*_R_) and UV spectrum for each peak were compared with those of the standards. The HPLC chromatogram was extracted at 305 nm. Three standard compounds including 5-(hydroxymethyl)furfural, *p*-coumaric acid, and *trans*-ferulic acid were obtained from Acros Organics (Geel, Belgium). Stock solutions (1.0 mg/mL) of single compounds were dissolved in methanol, and diluted to the required concentrations (50, 20, 10, 5, 2, 1 μg/mL) for creating respective calibration curves. They were injected onto HPLC system to generate calibration functions, which were calculated using peak area (*y*-axis) and concentration (*x*-axis). For quantitative analysis of SE sample, 200 mg of SE was extracted with 1 mL of methanol by sonication for 5 min, and 2 μL of extract solution was loaded onto HPLC system under the same condition. All injections were performed in triplicate.

### 4.3. Vascular Reactivity Study

Vascular reactivity study was performed using porcine coronary arteries as described previously [29]. Briefly, the left anterior descending coronary arteries of porcine heart (from the local slaughterhouse in Mokpo, Jeonnam, Korea) were dissected, cleaned of connective tissue, and cut into rings (4–5 mm in length) carefully. Then, porcine coronary artery rings were incubated in DMEM at 37 °C in a humidified atmosphere (95% air/5% CO_2_) for 18 h, in the absence (control) or presence of a high concentration of sodium (NaCl was added into the culture medium to obtain a final concentration of 160 mmol/L sodium), SE (containing 55.6% of NaCl; therefore, SE was added into the culture medium to obtain a final concentration of 160 mmol/L sodium), 5-(hydroxymethyl)furfural, *p*-coumaric acid, *trans*-ferulic acid and L-NAME (an inhibitor of eNOS, 0.1 mmol/L). After preincubation in different conditions, the rings were suspended in organ baths containing oxygenated (95% O_2_ and 5% CO_2_) Krebs bicarbonate solution (mmol/L; NaCl, 119; KCl, 4.7; KH_2_PO_4_, 1.18; MgSO_4_, 1.18; CaCl_2_, 1.25; NaHCO_3_, 25; and d-glucose, 11; pH 7.4, 37 °C) for the determination of changes in isometric tension. Following equilibration for 90 min under a resting tension of 5 g, the rings were contracted twice with KCl (80 mmol/L). Thereafter, the rings were pre-contracted with the thromboxane mimetic U46619 (1–60 nmol/L) to about 80% of the maximal contraction and the relaxation in response to bradykinin or indicated compounds was dose-dependent.

### 4.4. Animals and Experimental Design

Male SHRs and Sprague–Dawley (SD) rats were obtained from DreamBio (Gapyeong-gun, Gyeonggi-Do, Korea). All rats were kept at 25 °C in a room with a controlled atmosphere. Young (aged 6 weeks, 200 g ± 20% or less) SHR and age-matched SD rats were randomly divided into six groups (10 rats in each group): G1: vehicle control group (CTR–SD), G2: vehicle control group (CTR–SHR), G3: high salt (NaCl 800 mg/kg/day group, HS–SD), G4: high salt (NaCl 800 mg/kg/day group, HS–SHR), G5: SE 1400 mg/kg/day group (SE–SD), and G6: SE 1400 mg/kg/day group (SE–SHR). After 1 week of adaptive feeding with the common feed, a high quantity of edible salt and SE were administrated by gavage respectively for 6 weeks. The average daily food consumption of the rats was considered 20 g [30]; therefore, 4% NaCl would be equivalent to 800 mg/kg/day. SE contained 55.6% NaCl; therefore, we administered an equivalent quantity of SE (1400 mg/kg/day).

### 4.5. Body Weight and Feed Intake

Body weight and feed intake were measured at 1 week and 6 weeks, respectively, and changes in body weight and feed intake in each group were determined.

### 4.6. Kidney Index (KI) and Cardiac Index (CI) Analysis

The kidneys and hearts were maintained in cold physiological saline after scarifying and washing twice. Excess water was then removed using filter paper. Weights were determined, and the KI (kidney weight/body weight, KI) and CI (heart weight/body weight, CI) were calculated.

### 4.7. Determination of Tail Arterial Pressure

Tail arterial pressure was assessed by noninvasive tail-cuff plethysmography (BP-2000; Visitech Systems, Apex, NC, USA) after 1 week and 6 weeks. Before the administration of edible salt and SE, the rats were trained on the blood pressure device. One each day of blood pressure determination, 5 measurements were obtained and averaged for each rat.

### 4.8. Statistical Analysis

All data are expressed as mean ± standard error of the mean (SEM). Statistical analysis of the data was performed using the Student’s *t*-test or multiway ANOVA followed by Fisher’s protected least significant difference test where appropriate. A value of *p* < 0.05 was considered statistically significant.

## 5. Conclusions

In the present study, we investigated the effect of SE on vascular dysfunction and hypertension. SE did not induce dysfunction of the coronary artery even though it had a high salt content. HPLC analysis revealed that SE contained 5-(hydroxymethyl)furfural, *p*-coumaric acid, *trans*-ferulic acid, and other unknown compounds. Among them, *trans*-ferulic acid was responsible for the vascular protective effect of SE. In the in vivo study, high intake of purified salt increased blood pressure in normotensive and hypertensive rats; however, intake of SE, which has the same quantity of sodium as that in high edible salt, had little effect on blood pressure compared to high intake of purified edible salt. This result further suggests that SE, as a substitute for purified salt, might prevent the occurrence of hypertension and ameliorate hypertension status.

## Figures and Tables

**Table 1:** Kidney index (kidney weight/body weight, KI) and cardiac index (heart weight/body weight, CI) were calculated after six weeks of treatment with *Salicornia europaea* extracts (SE) and high edible salt.

Groups	KI (mg·g^−1^)	CI (mg·g^−1^)
CTR–SD	6.23 ± 0.31	3.18 ± 0.36
HS–SD	6.46 ± 0.38	2.86 ± 0.15
SE–SD	6.15 ± 0.43	2.72 ± 0.25
CTR–SHR	5.99 ± 0.31	3.62 ± 0.26 *
HS–SHR	6.19 ± 0.20	3.67 ± 0.28 *
SE–SHR	6.47 ± 0.43	3.96 ± 0.25 *

CTR–SD, control SD rat; HS–SD, high edible salt SD rat; SE–SD, *Salicornia europaea* extract SD rat; CTR–SHR, control SHR; HS–SHR, high edible salt SHR; SE–SHR, *Salicornia europaea* extract SHR. Values are expressed as mean ± SEM. “*” stand for *p* < 0.05 vs. CTR–SD.

## Abbreviations

SE	*Salicornia europaea* extracts
SBP	Systolic blood pressure
DBP	Diastolic blood pressure
MAP	Mean arterial pressure
CVD	Cardiovascular disease
WHO	World Health Organization
BP	Blood pressure
*t*_R_	Retention time
SHR	Spontaneously hypertensive rat
SD	Sprague–Dawley
KI	Kidney index
CI	Cardiac index
HPLC	High-pressure liquid chromatography
eNOS	Endothelial nitric oxide synthase
